# A novel optimized tiny YOLOv3 algorithm for the identification of objects in the lawn environment

**DOI:** 10.1038/s41598-022-19519-4

**Published:** 2022-09-06

**Authors:** Xinyan Wang, Feng Lv, Lei Li, Zhengyang Yi, Quan Jiang

**Affiliations:** School of Mechanical Engineering, Jiangsu University of Science and Technology, Zhenjiang, 212003 China

**Keywords:** Computer science, Mechanical engineering

## Abstract

Based on the problem of insufficient accuracy of the original tiny YOLOv3 algorithm for object detection in a lawn environment, an Optimized tiny YOLOv3 algorithm with less computation and higher accuracy is proposed. Three reasons affect the accuracy of the original tiny YOLOv3 algorithm for detecting objects in a lawn environment. First, the backbone of the original algorithm is composed of a stack of a single convolutional layer and a max-pooling layer, which results in insufficient ability to extract feature information of objects. An enhancement module is proposed to enhance the feature extraction capability of the shallow layers of the network. Second, the information of the shallow convolutional layers of the backbone is not fully used, which results in insufficient detection capability for small objects. Third, the deep part of the backbone uses a convolutional layer with an excessive number of channels, which results in a large amount of computation. A multi-resolution fusion module is proposed to enhance the information interaction capability of the deep and shallow layers of the network, and reduce the computation. To verify the accuracy of this Optimized tiny YOLOv3 algorithm, the algorithm was tested on the dataset containing trunk, spherical tree and person, and compared with the current research. The results show that the algorithm proposed in this paper improves the detection accuracy while reducing the calculation.

## Introduction

With the construction of the ecological environment, the lawn industry is developing rapidly, and the application of lawn machinery is becoming more and more extensive. The lightweight deep object detection technology based on deep learning can allow lawn machinery to automatically identify objects on the lawn, which can be used for obstacle avoidance or tree health etection to improve the degree of intelligence. Therefore, it is of great significance to the application of computer vision technology in the lawn environment.

With the rapid development of deep learning techniques based on convolutional neural networks^1–3^, significant achievements have been made in the field of object detection. Generally, object detection algorithms can be classified into two types: two-stage and one-stage algorithms. The first stage of the two-stage algorithm involves extracting the depth features of the image through a RPN (region proposal network) to generate candidate regions. The second stage selects the candidates through the object detection network. Subsequently, the regions are classified and located. The algorithms of this type exhibit a high accuracy rate, but the detection speed is slow; examples of two-stage algorithms include R-CNN^4^ (region convolutional neural networks), fast R-CNN^5^, faster R-CNN^6^ and SPP^7^. The slow detection rate is attributed to the two stages in the algorithm.

Conversely, the one-stage algorithm directly predicts the category and position of the object through the backbone network without the RPN network. Although the detection accuracy is reduced, the detection speed is significantly improved. Commonly used algorithms are the SSD^8,9^ (single shot multibox detector) series and YOLO^10–12^ (you only look once) series. The YOLO algorithm solves the object detection task as a regression problem. It can directly process the input image and output the result. At present, it is one of the fastest detection network architectures. However, the aforementioned algorithms rely on high-performance computing hardware. Due to the limited computing ability of embedded devices, it is impossible to deploy the aforementioned large neural networks.

Since the efficient execution of deep neural networks on mobile devices has become part of mainstream research^13–15^, the YOLO series is being developed further; a tiny YOLO series version that is more suitable for deployment on mobile devices has been designed. Tiny YOLOv3 reduces a large number of convolutional layers in the YOLO series and reduces the size of the network, thereby reducing the hardware computing power requirements and increasing the detection speed. However, it reduces the detection accuracy and results in a high missing detection rate. Zhang^16^ improved the ability to detect pedestrians by merely adding convolutional layers to the tiny YOLOv3 network. Gai^17^ proposed the improved tiny YOLOv3 for real-time object detection by adding convolutional layers. He^18^ proposed the TF-YOLO to increase the detection accuracy of the tiny YOLOv3 network by adding one YOLO layer. Wu^19^ proposed a light YOLOv3 network to detect apples using a residual block composed of depthwise separable convolutions. Liu^20^ increased the detection accuracy of the tiny YOLOv3 network by adding one YOLO layer.

The original tiny YOLOv3 algorithm has low detection accuracy on our lawn environment target dataset. (1) We design an enhancement module to improve the detection accuracy of backbone. (2) We design a multi-resolution fusion module to enhance the information interaction capability inside the backbone and reduce the amount of calculation. (3) On this basis, the Optimized tiny YOLOv3 algorithm is proposed. Comparison experiment with the current three lightweight YOLO algorithms shows that the algorithm proposed in this paper is superior to the others in terms of accuracy and lightweight degree.

## Optimized tiny YOLOv3 algorithm network

### Loss function

To improve the speed and convergence of the bounding box regression, the CIoU^21^ (complete intersection over union) loss function is adopted as the loss function,

1 $$ L_{{{\text{CIoU}}}} = 1 - {\text{IoU}} + R_{{{\text{CIoU}}}} . $$

**Figure 1:**
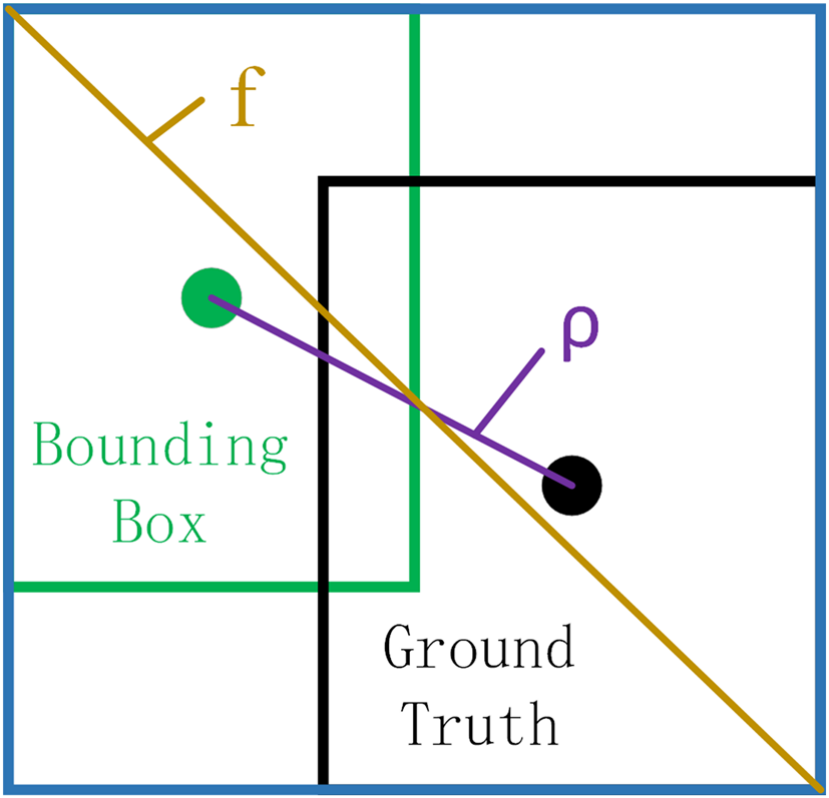
Theory of CIoU loss regression of bounding box.

R_CIoU_ is the

2 $$ R_{{{\text{CIoU}}}} = \frac{{\rho^{2} \left( {b,b^{{{\text{gt}}}} } \right)}}{{f^{2} }} + \alpha v, $$

3 $$ \alpha = \frac{v}{{(1 - {\text{IoU}}) + v}}, $$

4 $$ v = \frac{4}{{\pi^{2} }}\left( {\arctan \frac{{w^{{{\text{gt}}}} }}{{h^{{{\text{gt}}}} }} - \arctan \frac{w}{h}} \right)^{2} , $$

where, *α* is the weight function; *v* is the similarity parameter of the aspect ratio; *b* and *b*^*gt*^ represent the center points of the bounding box and the ground truth, respectively; *ρ* is the Euclidean distance between the two center points; *f* is the diagonal length of the smallest rectangle that can contain both the bounding box and the ground truth, as shown in Fig. 1.

### Enhancement module

BFLOPs (billion float operations per seconds) is the number of floating-point operations of the convolutional neural network, and it is a parameter to measure amount of calculation. The calculation equation is as follows,

5 $$ Calculation = \frac{{2HWK_{h} K_{w} C_{in} C_{out} }}{{10^{9} }}, $$

where, *H*, *W* and *C*_*out*_ are the width, height and number of channels of the output feature map, respectively. *C*_*in*_ is the number of channels of the input feature map. *K*_*h*_ and *K*_*w*_ are the sizes of the convolution kernel.

**Figure 2:**
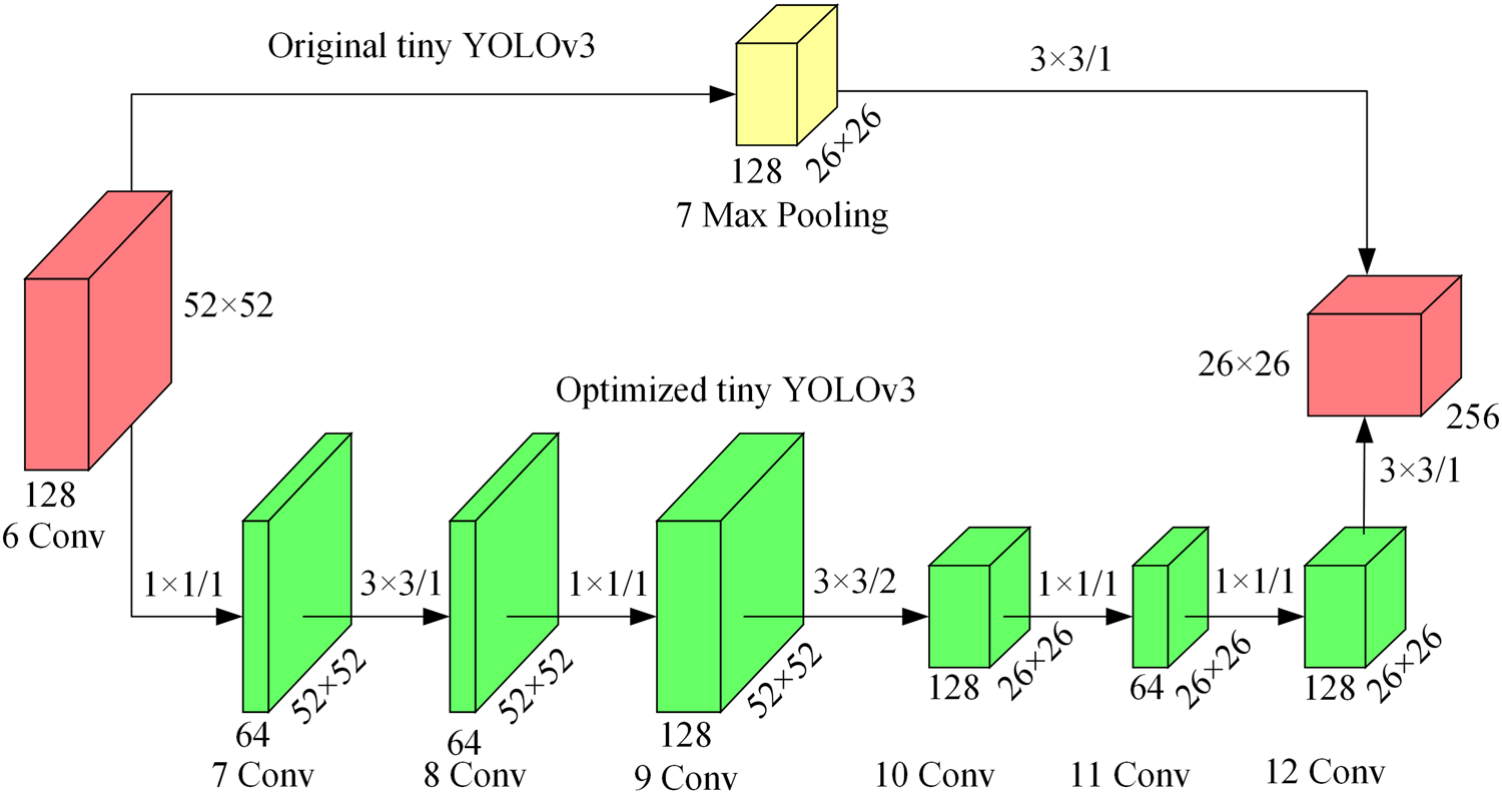
Enhancement module.

In order to solve the problem of insufficient utilization of the shallow feature information of the network, the backbone is improved, and an enhancement module is proposed to strengthen feature extraction, as shown in Fig. 2.

The convolutional layers of 3 × 3 and 1 × 1 are used to enhance feature extraction and fusion for the feature map output from the 6th layer of the network, instead of using the max pooling to reduce the dimension. The point convolution is used for cross-channel fusion and the number of channels is compressed from 128 to 64, which reduces the amount of computation. A 3 × 3 convolutional layer is used for feature extraction, and the number of output channels is still 64. Finally, the point convolution is used to expand the number of channels to 128. Different from the 7th layer in the original backbone that uses a max-pooling layer to reduce the size, a 3 × 3 convolutional layer with stride 2 is used to extract feature information while reducing the dimension. A point convolutional layer is used to compress the channel of the feature map to 64, and then expand it to 128, compress the invalid feature map with little calculation, and generate effective feature maps. A 3 × 3 convolutional layer is used to extract feature maps, resulting in the same output as the original network. It can be seen from Eq. (5) that the calculation amount of the enhancement module is 0.9BFLOPs, which is only 0.1BFLOPs increased compared to the original tiny YOLOv3.

### Multi-resolution fusion module

The max-pooling layer is widely used by the backbone of the original tiny YOLOv3, resulting in the loss of a large amount of semantic information during the downsampling process and the missed detection of small objects. In order to solve the above problems and reduce the increased amount of calculation in the enhancement module, a multi-resolution fusion module is proposed.

**Figure 3:**
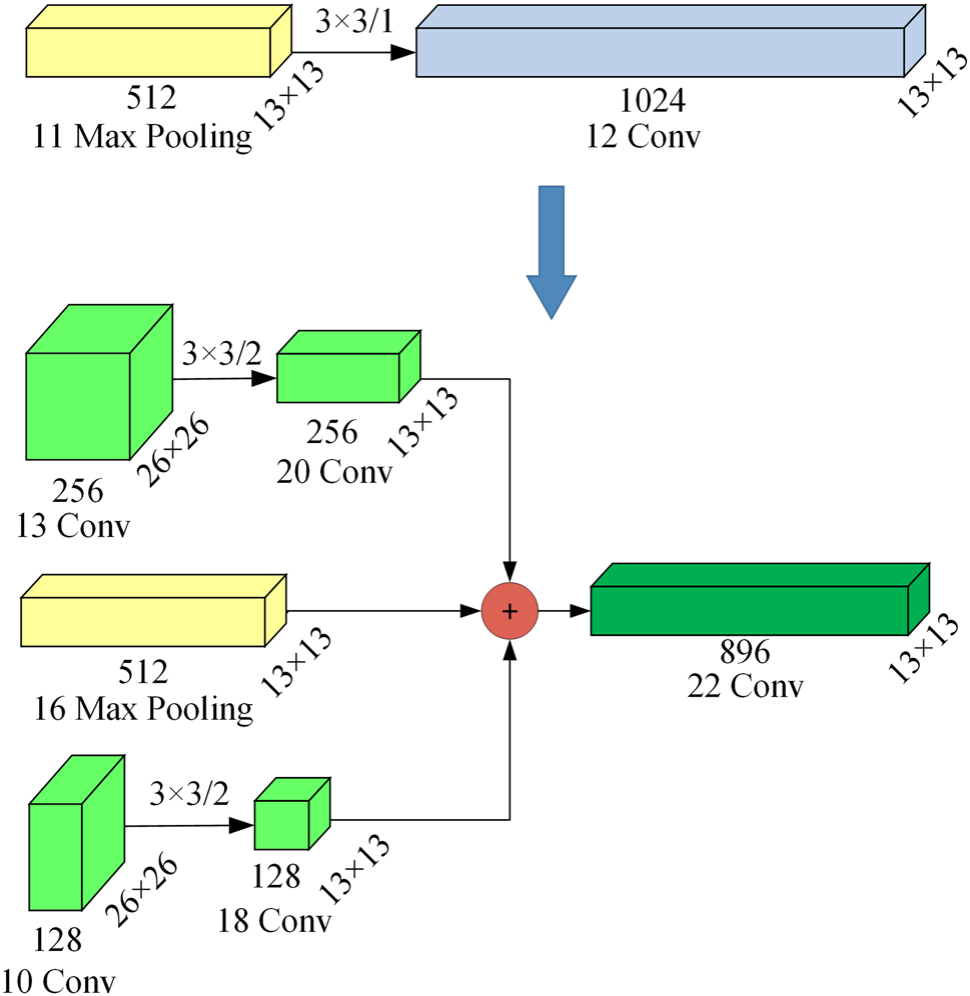
Multi-resolution fusion module.

The last layer in the original Tiny YOLOv3 backbone is at the top of the FPN^22^ (feature pyramid networks) network. The resolution of the output feature map is 13 × 13 and the number of channels is 1024, which requires a huge amount of calculation. A multi-resolution fusion module is used to replace this layer. Since the backbone contains few convolutional layers, only making full use of the convolutional feature maps of the shallow layers of the network can improve the detection accuracy. The multi-resolution fusion module is shown in Fig. 3.

In the first part, a convolutional layer of size 3 × 3 and stride 2 is used to extract and reduce the dimension of the feature map output by the 13th layer of the backbone after adding the enhancement module. The second part is the feature map of the 16th layer output of the backbone after adding the enhancement module. In the third part, a convolutional layer of size 3 × 3 and stride 2 is used to extract and reduce the dimension of the feature map output by the 10th layer of the backbone after adding the enhancement module. Finally, the three parts are spliced with concat to form a feature map of 13 × 13 × 896.

It can be seen from Eq. (5) that the calculation amount of the last layer of the original tiny YOLOv3 is 1.60BFLOPs, and the calculation amount of the multi-resolution fusion module is 0.25BFLOPs. It can be seen that the multi-resolution fusion module not only further utilizes the information of the shallow layers of the backbone, but also reduces the amount of calculation by 1.35 BFLOPs.

### Network structure

**Figure 4:**
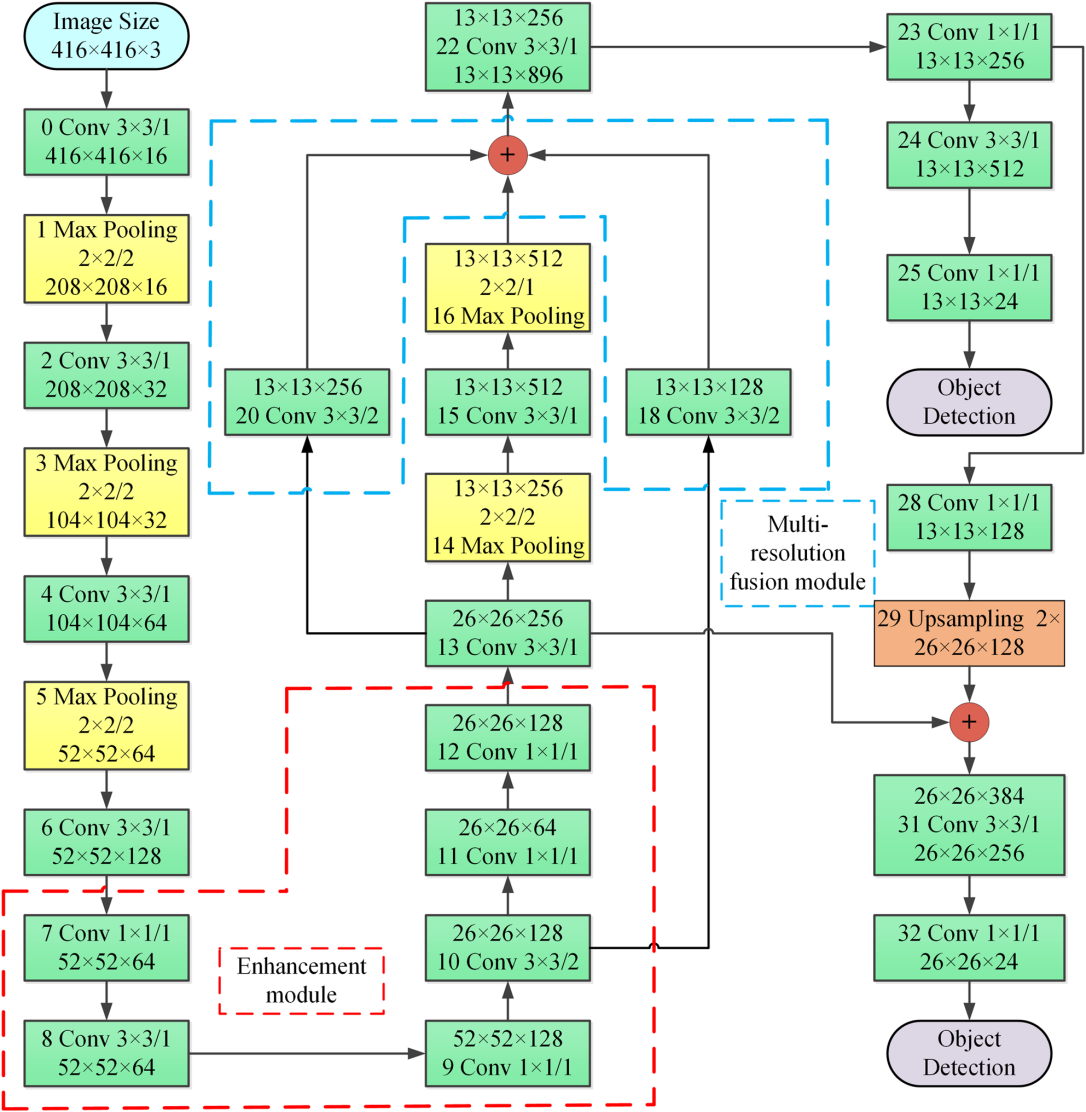
Optimized tiny YOLOv3 algorithm network.

The whole Optimized network is shown in Fig. 4.

The total calculation amount of the original tiny YOLOv3 is 5.45BFLOPs, and the total calculation amount of Optimized tiny YOLOv3 using enhancement module and multi-resolution fusion module is 5.25BFLOPs, a reduction of 3.7%.

## Materials and methods

### Dataset

**Figure 5:**
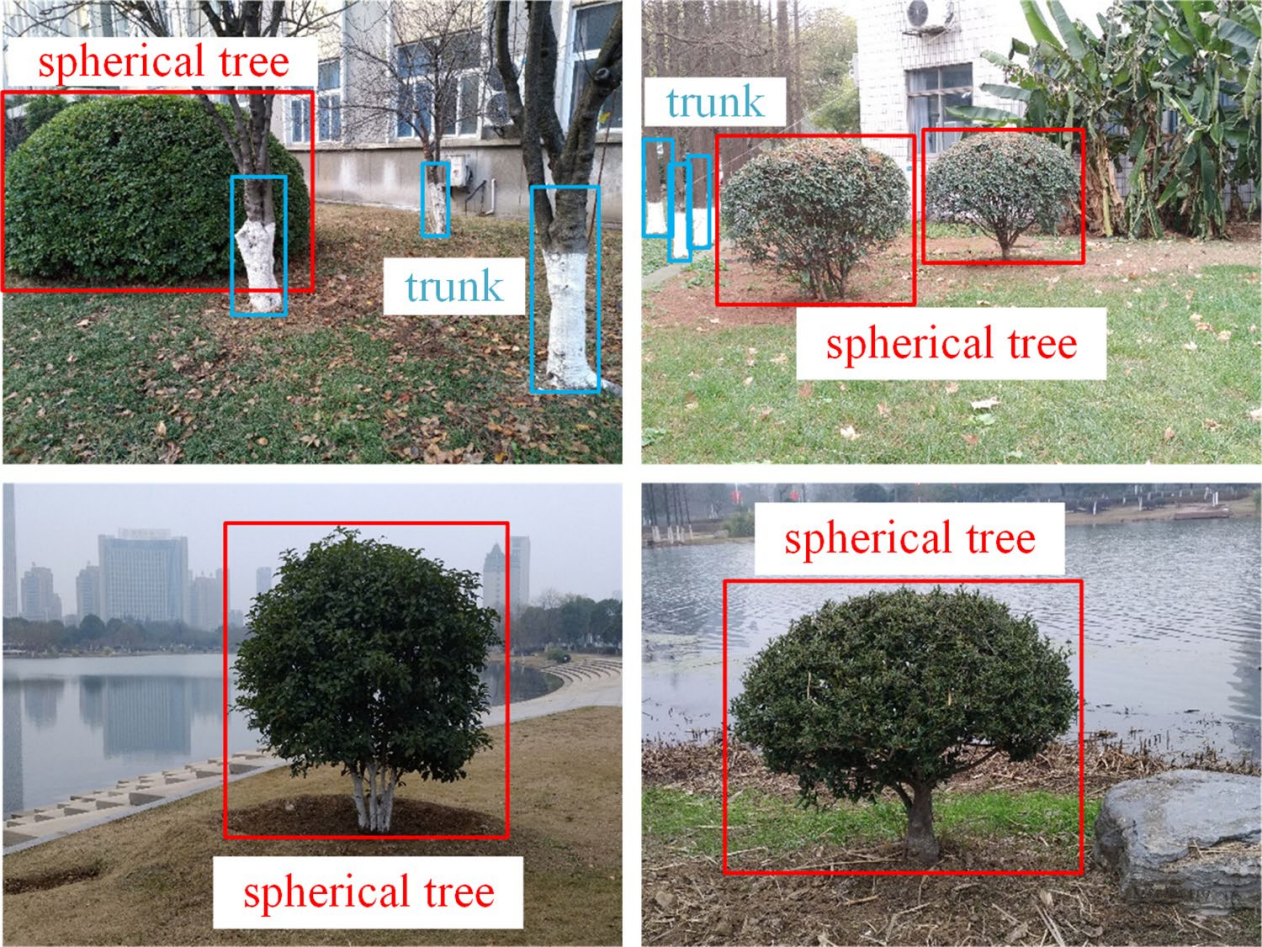
Trunk and spherical tree in dataset.

The lawn environment object dataset was made to train the algorithm. The original static obstacles in the lawn environment mainly comprise trunks and spherical trees, whereas the dynamic obstacles mainly comprise people. There exists no publicly available dataset that meets the requirements of this paper. Therefore, to verify the optimized algorithm network, it is necessary to create the dataset. The developed dataset has three main categories, namely, trunk, spherical tree, and person. Trunks and spherical trees were taken from the lawn environment field shooting, a total of 8059, including 7922 trunk samples and 567 spherical tree samples, and the picture size was 564 × 422 × 3. Trunk and spherical tree are shown in Fig. 5.

**Figure 6:**
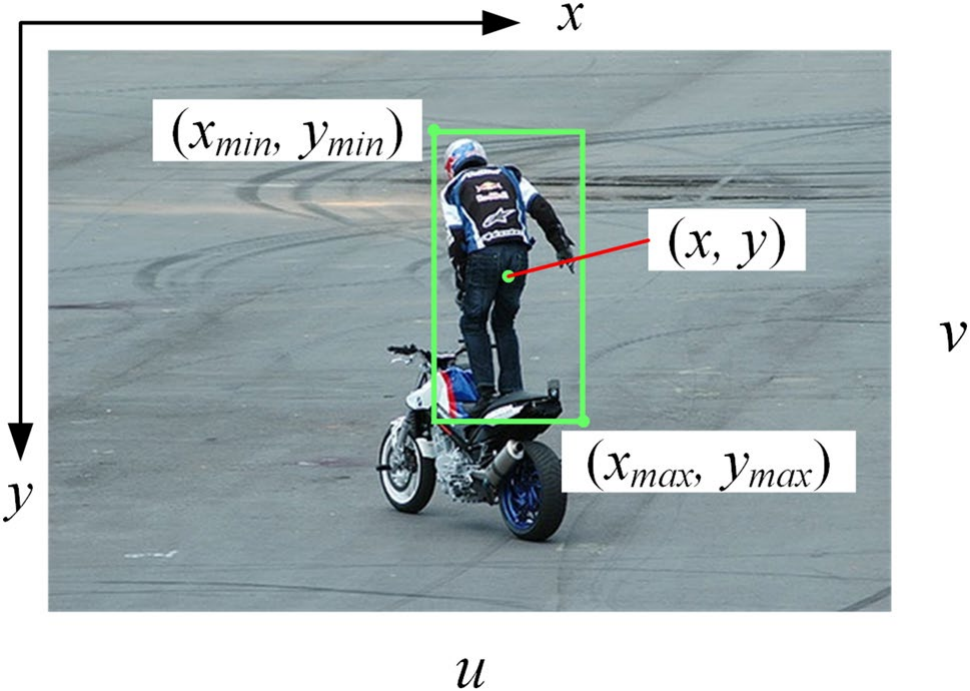
Annotation parameters of an object in the coordinates.

The person dataset was derived from all the pictures in the PASCAL VOC 2007 covering the person category, with 4012 pictures. The entire dataset has 12,071 pictures. To accelerate the network convergence speed and prevent gradient explosion, the labeled data are normalized, and the format of the normalized labeled data is

$$ \begin{array}{*{20}l} {class\_id} \hfill & x \hfill & y \hfill & w \hfill & h \hfill \\ \end{array} , $$

where, *class_id* is the object category, trunk is 0, spherical tree is 1, and person is 2. *x* and *y* are the coordinates of the center point, and *w* and *h* denote the width and height of the normalized object bounding box, respectively.

6 $$ x = \left( {x_{\max } + x_{\min } } \right)/2u, $$

7 $$ y = (y_{\max } + y_{\min } )/2v, $$

8 $$ w = (x_{\max } - x_{\min } )/u, $$

9 $$ h = (y_{\max } - y_{\min } )/v, $$

where, *x*_*min*_ and *y*_*min*_ are the coordinates of the upper left corner of the target bounding box, *x*_*max*_ and *y*_*max*_ are the coordinates of the lower right corner of the object bounding box, and *u* and *v* are the width and height of the picture, respectively, as shown in Fig. 6.

The dataset was randomly divided, and the number of images in the training set, validation set and testing set are 7727, 1932 and 2412, respectively. The anchor boxes are clustered using the k-means++ algorithm, an extension of k-means^23^, and the sizes are (12, 48), (30, 99), (49, 206), (110, 149), (94, 331), and (243, 329).

### Algorithm training

In terms of the network training platform, the operating system is Windows 10, the CPU is an Intel i7-9700KF with 3.6 GHz clock speed, the memory size is 16 GB, the GPU is a NVIDIA GEFORCE RTX 2070 Super with 8 GB memory size, the deep learning framework is AlexeyAB-Darknet, and the compilation environment is Visual Studio 2015 with C/C++ language.

The total number of training iterations of the Optimized tiny YOLOv3 algorithm was 25,000, the initial learning rate was set to 0.00261, and when it was trained to 15,000 rounds and 25,000 rounds, learning rate was reduced to 10%. The decay value was set to 0.0005. During the training process, the images were rotated and the hue and saturation were changed to prevent overfitting. The loss function uses CIoU, and the type of non-maximum suppression is greedy-NMS (greedy non maximum suppression). The Optimized tiny YOLOv3 algorithm is compared with original tiny YOLOv3, Improved tiny YOLOv3 and TF-YOLO in terms of loss and mAP (mean average precision) on the lawn environment object dataset.

**Figure 7:**
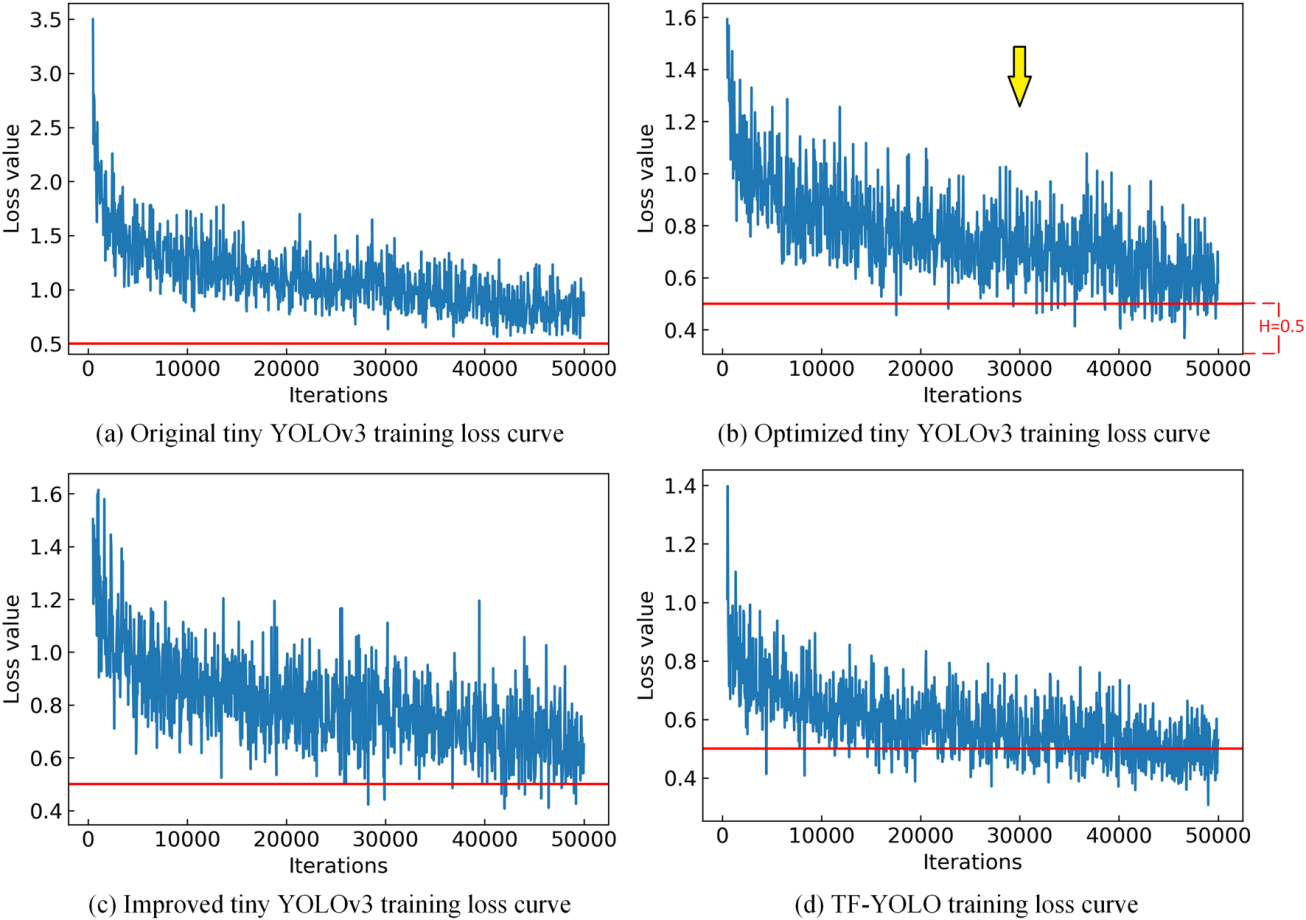
Loss function curves.

The curve of the loss function value and the number of training iterations of each algorithm during the training process is shown in Fig. 7. The red line in the figure is where the loss function value is 0.5, and the loss function value below 0.5 can be considered to have good detection performance.

The higher the relative height of the red line from the bottom horizontal axis, the better the convergence of the algorithm. In Fig. 7, TF-YOLO has the highest convergence, followed by Optimized tiny YOLOv3. Compared with original tiny YOLOv3, the algorithm proposed in this paper has a significant improvement in convergence.

**Figure 8:**
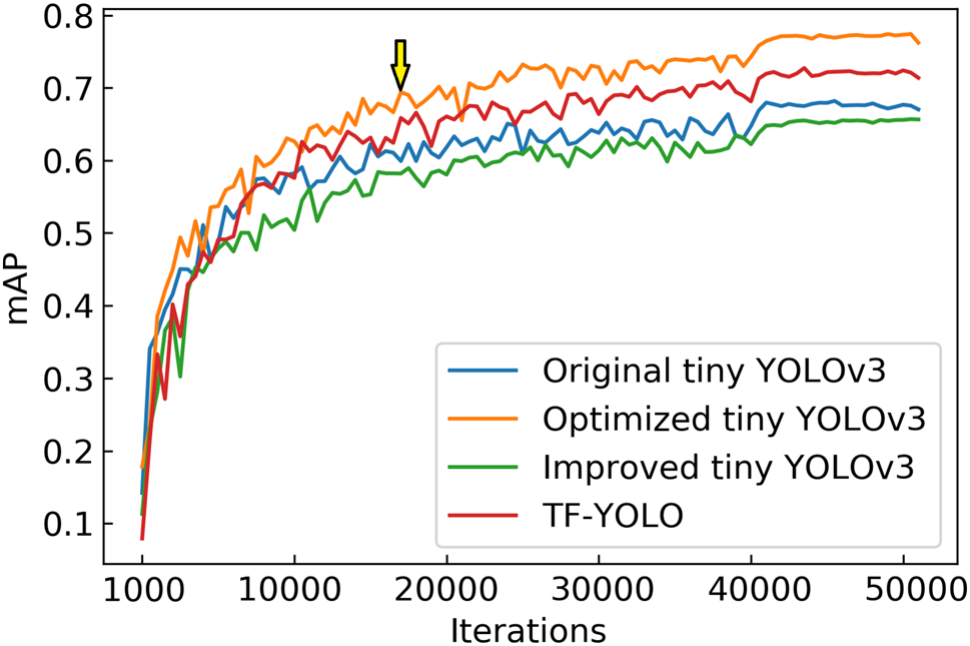
mAP curves.

During the training process, the mAP curve is shown in the Fig. 8.

In Fig. 8, Optimized tiny YOLOv3 has the highest mAP value on the validation set compared to all other algorithms, and has the best training effect on the training set.

## Results and discussion

**Figure 9:**
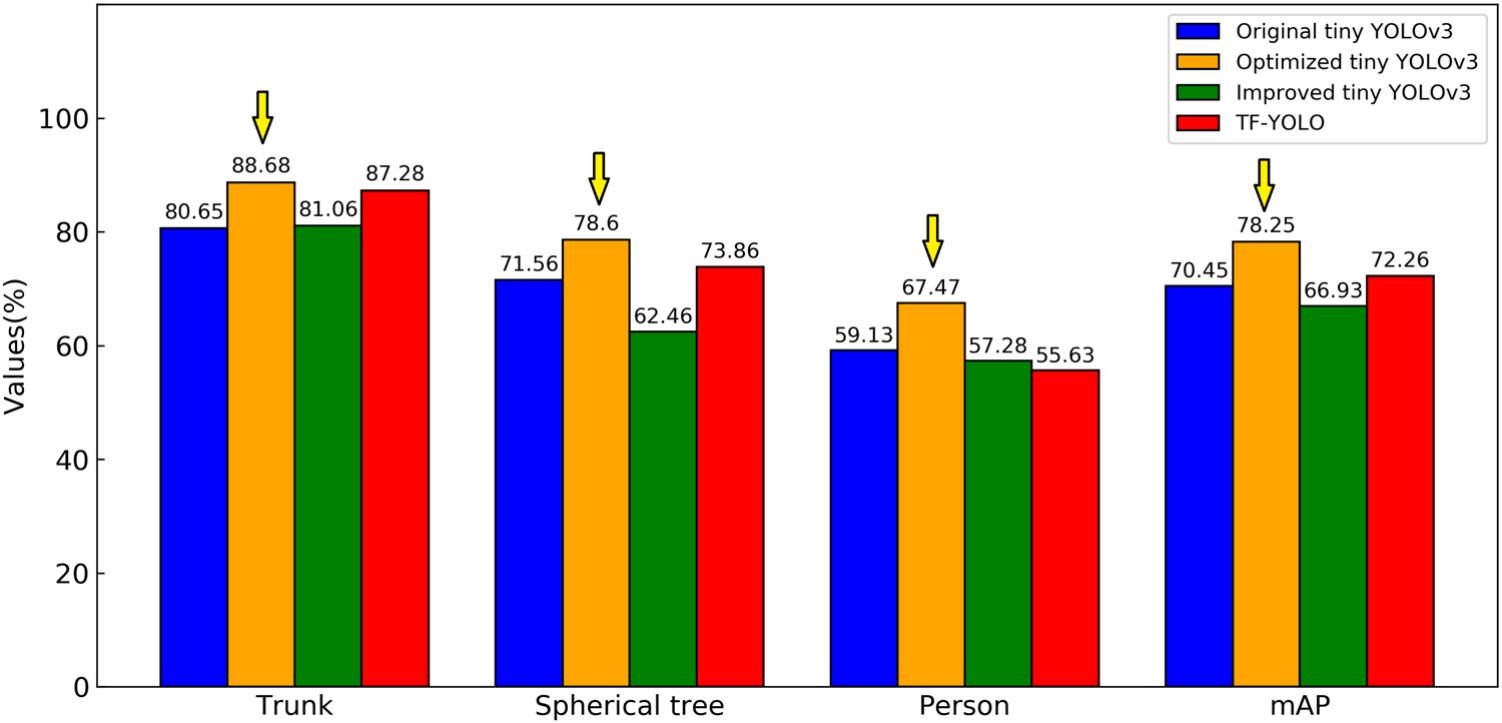
AP and mAP values on the testing set.

The AP (average precision) of each class and the mAP of all classes are used to evaluate the accuracy of algorithms, and the amount of calculation is used to evaluate the lightweight degree. The four algorithms involved in training are tested on the testing set, and the AP of each class and the overall mAP are counted, as shown in Fig. 9.

It can be seen from Fig. 9 that the detection accuracy of the Optimized tiny YOLOv3 algorithm proposed in this paper for trunk, spherical tree and person is improved by 8.03%, 7.04% and 8.34% respectively compared with the original tiny YOLOv3. The mAP value has improved significantly, increasing by 7.8%. The data tested on the testing set of the algorithm proposed in this paper show that it has the best performance in terms of detection accuracy.

**Figure 10:**
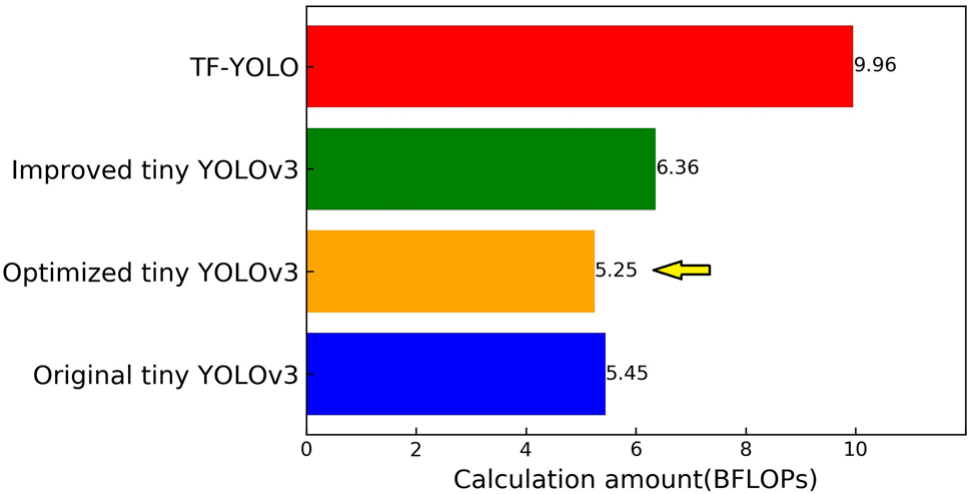
Calculation amount of each algorithm.

The calculation amount of each algorithm is shown in Fig. 10.

It can be seen from Fig. 10 that the Optimized tiny YOLOv3 algorithm proposed in this paper has the smallest amount of computation. Compared with original tiny YOLOv3, amount of calculation is reduced by 0.2BFLOPs. The calculation amount of Optimized tiny YOLOv3 is much smaller than the TF-YOLO algorithm.

Compared with the original tiny YOLOv3 algorithm, the Optimized tiny YOLOv3 algorithm proposed in this paper greatly improves the detection accuracy under the condition that the lightweight degree is slightly improved, and the accuracy and lightweight degree are better than Improved tiny YOLOv3 and TF-YOLO.

## Conclusions

In this work, we explore the application of deep learning-based object detection technology in the lawn environment, providing a research example for agricultural intelligence.

A dataset containing trunk, spherical tree and person is specially made for the lawn environment, which provides dataset support for subsequent neural network-based target detection algorithms.

Aiming at the problem of insufficient backbone extraction capability of the original tiny YOLOv3 algorithm, an enhancement module is proposed to enhance the feature extraction capability. A multi-resolution fusion module is proposed for the poor detection of small objects by the backbone to strengthen the information interaction between the deep and shallow convolutional layers.

Based on the enhancement module and the multi-resolution fusion module, Optimized tiny YOLOv3 algorithm is proposed. Experiment on the dataset shows that the algorithm proposed in this paper not only reduces the amount of calculation but also greatly improves the detection accuracy.

## Data Availability

All data generated or analysed during this study are included in this published article.

## References

[1] Krizhevsky A, Sutskever I, Hinton G (2012). ImageNet classification with deep convolutional neural networks. Adv. Neural Inf. Process. Syst..

[2] Lecun Y, Bengio Y, Hinton G (2015). Deep learning. Nature.

[3] Chollet, F. Xception: Deep learning with depthwise separable convolutions. In *IEEE Conference on Computer Vision and Pattern Recognition*, 1800–1807 (2017).

[4] Zhang N, Donahue J, Girshick R, Darrell T (2014). Part-based r-cnns for fine-grained category detection. Lect. Notes Comput. Sci..

[5] Girshick, R. Fast r-cnn. In *IEEE International Conference on Computer Vision*, 1440–1448 (2015).

[6] Ren S, He K, Girshick R, Sun J (2017). Faster r-cnn: Towards real-time object detection with region proposal networks. IEEE Trans. Pattern Anal. Mach. Intell..

[7] He K, Zhang X, Ren S, Sun J (2015). Spatial pyramid pooling in deep convolutional networks for visual recognition. IEEE Trans. Pattern Anal. Mach. Intell..

[8] Liu W (2016). SSD: Single shot multibox detector. Lect. Notes Comput. Sci..

[9] Fu, C. Y., Liu, W., Ranga, A., Tyagi, A. & Berg, A. C. Dssd: Deconvolutional single shot detector. In *IEEE Conference on Computer Vision and Pattern Recognition* (2017).

[10] Redmon, J., Divvala, S., Girshick, R. & Farhadi, A. You only look once: Unified, real-time object detection. In *IEEE Conference on Computer Vision and Pattern Recognition*, Vol. 2016 (2016).

[11] Redmon, J. & Farhadi, A. YOLO9000: Better, faster, stronger. In *IEEE Conference on Computer Vision and Pattern Recognition*, 6517–6525 (2017).

[12] Redmon, J. & Farhadi, A. YOLOv3: An incremental improvement. In *IEEE Conference on Computer Vision and Pattern Recognition* (2018).

[13] Wu, B., Wan, A., Iandola, F., Jin, P. H. & Keutzer, K. Squeezedet: Unified, small, low power fully convolutional neural networks for real-time object detection for autonomous driving. In *IEEE Conference on Computer Vision and Pattern Recognition Workshops*, 446–454 (2017).

[14] Howard, A. G. *et al*. MobileNets: Efficient convolutional neural networks for mobile vision applications. In *IEEE Conference on Computer Vision and Pattern Recognition* (2017).

[15] Shafiee MJ, Chywl B, Li F (2017). Fast YOLO: A fast you only look once system for real-time embedded object detection in video. J. Comput. Vis. Imaging Syst..

[16] Zhang Y, Shen Y, Zhang J (2019). An improved tiny-YOLOv3 pedestrian detection algorithm. Optik.

[17] Gai W, Liu Y, Zhang J, Jing G (2021). An improved tiny YOLOv3 for real-time object detection. Syst. Sci. Control Eng..

[18] He W, Huang Z, Wei Z, Li C, Guo B (2019). TF-YOLO: An improved incremental network for real-time object detection. Appl. Sci..

[19] Wu X, Qi Z, Wang L, Yang J, Xia X (2019). Apple detection method based on light-YOLOv3 convolutional neural network. Trans. Chin. Soc. Agric. Mach..

[20] Liu J, Hou S, Zhang K, Zhang R, Hu C (2019). Real-time vehicle detection and tracking based on enhanced tiny YOLOv3 algorithm. Trans. Chin. Soc. Agric. Eng..

[21] Zheng Z (2019). Distance-IoU loss: Faster and better learning for bounding box regression. Proc. AAAI Conf. Artif. Intell..

[22] Lin, T. Y., Piotr, D., Girshick, R. & He, K. Feature pyramid networks for object detection. In *IEEE Conference on Computer Vision and Pattern Recognition*, 936–944 (2017).

[23] Hartigan JA, Wong MA (1979). A k-means clustering algorithm. J. R. Stat. Soc..

